# Non-healing trophic ulcer in leprosy: a case of failure at all levels of prevention

**DOI:** 10.11604/pamj.2023.45.50.39126

**Published:** 2023-05-22

**Authors:** Himabindu Reddy, Abhishek Joshi

**Affiliations:** 1 Jawaharlal Nehru Medical College, Sawangi (Meghe), Wardha, Maharashtra, India

**Keywords:** Neglected tropical diseases, leprosy, trophic ulcer

## Image in medicine

Hansen's disease, one of the neglected tropical diseases, is on the WHO agenda as ‘Towards zero leprosy strategy,’ aiming to eliminate disease disability and discrimination associated with leprosy by 2030. This year marks the 150^th^ anniversary of the discovery of the causative organism *‘Mycobacterium Leprae’*. To achieve this target, it is crucial to target all angles of the epidemiological triad, i.e. to not just cure existing cases but also prevent new incidences or transmission of Leprosy. The image shows the right foot of a 65-year-old lady, with a case of multibacillary leprosy on multidrug therapy (MDT) for 1 year. She first noticed a wound on the plantar aspect of her foot (two toes autoamputated) six months back (A) for which she only took some herbal concoction from a local non-medical healer. She had been abandoned by her family over a year ago upon her leprosy diagnosis, so her predicament remained neglected. We came across this case during a health camp in the village. Upon examination, the wound was determined to be an 8cm x 5cm x 2cm ulceroproliferative lesion on the lateral aspect of the foot, with irregular margins, raised rolled-over edges, with slough and surrounding induration. It bled on touch (B) but was not associated with pain or tenderness. Loss of sensation and pain due to Hansen's, forgoing appropriate footwear due to lack of awareness, stigma towards leprosy patients and long distance to leprosy center (> 20km) caused a simple wound to progress to more than half her foot, thus debilitating her.

**Figure 1:**
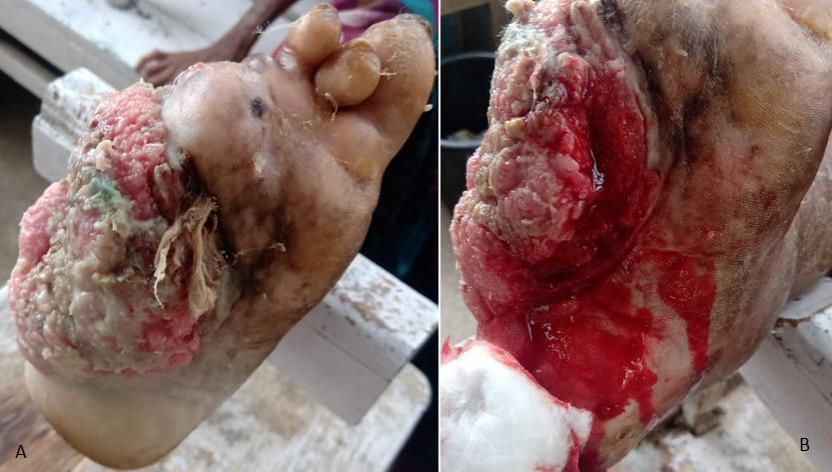
trophic ulcer in leprosy; A) auto-amputation of toes, Hansen's disease; B) bleeding + on touching

